# Epidemiological survey of PRRS and genetic variation analysis of the ORF5 gene in Shandong Province, 2020–2021

**DOI:** 10.3389/fvets.2022.987667

**Published:** 2022-09-15

**Authors:** Peixun Li, Yesheng Shen, Tailong Wang, Jing Li, Yan Li, Yiran Zhao, Sidang Liu, Baoquan Li, Mengda Liu, Fanliang Meng

**Affiliations:** ^1^College of Animal Medicine, Shandong Agricultural University, Taian, China; ^2^College of Veterinary Medicine, Huazhong Agricultural University, Wuhan, China; ^3^Division of Zoonoses Surveillance, China Animal Health and Epidemiology Center, Qingdao, China; ^4^Huayun (Shandong) Inspection and Quarantine Service Co., Ltd, Taian, China

**Keywords:** PRRSV, GP5, epidemiology, genetic evolutionary analysis, prevention and control

## Abstract

Since the rise of porcine reproductive and respiratory syndrome virus (PRRSV) in China, mutations have occurred regularly. In particular, the emergence of HP-PRRSV has significantly improved the pathogenicity of PRRSV. It has brought huge economic losses to the Chinese pig farming industry. To understand the current prevalence and evolution of PRRSV in Shandong Province, 1,344 samples suspected of having PRRSV were collected from local hog farms of different sizes. Genetic variation in the isolated PRRSV ORF5 gene was analyzed using the RT-PCR method. The results showed that the detection rate of PRRSV in the collected samples was 25.44%. The predominant strain of PRRSV in Shandong Province is still NADC30-like. However, it cannot be ignored that NADC34-like is also starting to become a prevalent strain. Mutations in ORF5 amino acids 13, 151 and neutralizing epitope (aa36-aa52) in some isolates can cause changes in virulence and ability to escape immunity. This study enriches the epidemiological data on PRRSV in Shandong Province, China. It provides an important reference for the development of new vaccines and for the prevention and control of PRRSV.

## Introduction

Porcine reproductive and respiratory syndrome (PRRS) is a highly contagious disease caused by the porcine reproductive and respiratory syndrome virus (PRRSV) (1). It is known as “blue ear disease” because it often leads to bluish purple ears in diseased pigs (2). In affected pigs, the disease primarily causes spontaneous abortion in late pregnancy, as well as stillbirths, mummified fetuses, or weak piglets. It also causes congenital dysplasia in piglets, respiratory distress, interstitial pneumonia and suppressed immune function. Moreover, it is often seen in mixed infections with other pathogens (3–5). PRRSV is currently one of the most serious pathogens posing a threat to global swine production. After beginning in Europe and the Americas in the 1990s, it spread across the globe (6, 7). The virus was first isolated in China in 1996 (5), and since then PRRSV has become widely prevalent there. A highly pathogenic strain of PRRSV emerged in China in 2006 and became the dominant epidemic strain (8–12). In 2012, in Henan Province, China, Zhou et al. (13) discovered for the first time a NADC30-like highly homologous strain with 131 aa discontinuous deletions in the nsp2 gene. In 2013–2015, NADC30-like strains were reported in many provinces in China (14). The high frequency of NADC30 recombination makes the prevention and control of PRRS extremely difficult (15–17). In 2017, the NADC34 strain was isolated for the first time in China (18). NADC34-like PRRSV is now mildly or moderately pathogenic to piglets (19–21). It primarily affects sows, often leading to severe spontaneous abortions among pregnant sows (19). NADC34-like PRRSV has become widespread in several provinces. The prevalence of NADC34 strains has been observed in 10 provinces and cities, including Heilongjiang, Liaoning, Jilin, Jiangsu, Henan, Hebei and Shandong (22–24). This makes it very necessary to monitor PRRSV and understand its prevalence in Shandong Province.

The genome of PRRSV is approximately 15 kb long, forms a cap structure at the 5′ end during mRNA processing, and has a Poly-A tail structure at the 3′ end (25). The structural proteins of this virus are GP2a, GP3, GP4, GP5, M, E, and N. Among these, GP5 and M proteins are the main envelope proteins of PRRSV (26). GP5 protein is the most variable protein in PRRSV. In addition, GP5 contains glycosylation sites that help recognize cell receptors and neutralize viruses. GP5 is also the main protein that promotes the production of neutralizing antibodies in the body. The rapid mutation and the high recombination frequency of PRRSV stimulate the prevalence of PRRS and exacerbate the difficulty of PRRS prevention and control (27). Therefore, the GP5 protein, as an extremely important PRRSV protein, has become an important indicator in the identification and analysis of PRRSV. To understand the latest epidemiological situation and epidemic strain types of PRRSV in 2020–2021, an experiment was conducted. In this experiment, clinical cases of suspected PRRSV infections were collected from swine farms of varying sizes in Shandong Province. The pathogens were detected by RT-PCR and were sequenced and analyzed for the GP5 protein gene.

## Materials and methods

### Sample collection

In this study, suspected cases of PRRS were collected from pig farms of different sizes in all cities of Shandong Province in 2020–2021. Blood and nasal cotton swabs were mainly collected from affected pigs, while lymph nodes and lung tissues were collected from dead pigs.

### Sample handling

The lymph nodes and pulmonary tissues were collected aseptically excised and crushed in a suspension. The blood was mixed with the corresponding nasal cotton swab. All processed samples were centrifuged, and the supernatant was sucked out to extract the total RNA for the RT-PCR test. Samples containing the low-viral target bands with content were inoculated into Marc-145 cells and PAM cells for blind transmission in three generations. The virus fluid was collected for the RT-PCR test.

### Viral RNA extraction

The viral solution was added to the RNA isolater, and the total RNA was extracted according to the instructions of RNA isolater Total RNA Extraction Reagent (Vazyme).

### RT-PCR assay

With the use of the HiScript III 1st Strand cDNA Synthesis Kit (Vazyme), the resulting total RNA was reverse transcribed into the cDNA in accordance with the instructions. PCR amplification was performed using the primers in Table 1. The results were observed through agarose gel electrophoresis under a gel system imager.

**Table 1:** List of primers used in this study.

Names*	**Primer sequence (5^′^–3^′^)**	**Length (bp)**
GP5-F	GGGCAACCGTTTTAGCCTGTC	710
GP5-R	GAACGCCAAAAGCACCTTCTG	

^*^F represents forward PCR primer; R represents reverse PCR primer.

### GP5 gene sequencing and analysis

All samples with positive RT-PCR findings were sequenced for the ORF5 gene. The sequences related to PRRSV Sublineage 1.5, Sublineage 1.8, Lineage 3, Lineage 5, and Lineage 8 were downloaded from the NCBI database as reference strains. These were analyzed using MEGA X and MegAlign.

### Recombination analysis

In this study, the full-length genome of SDHY-DZ037 was sequenced for recombination analysis. Alignment was screened using RDP4, implementing the RDP (28), GENECONV (29), Bootscan (30), Chimaera (31), SiScan (32), MaxChi (33), and 3Seq (34) algorithms. At least four of the above methods can identify a recombination event. In addition, if the breakpoint region of the recombination event is larger than 100 nt, the region can be regarded as a recombination region. To confirm these presumed recombinant events, we generated a series of phylogenetic trees for each sequence region identified during the analysis (35). For each region, evolutionary analysis of maximum likelihood was performed in MEGA X. To visualize the recombinant signal and inferred breakpoint locations, a similarity analysis between the presumptive recombinant sequences and the parental lineages was implemented in SIMPLOT v3.5.1 (36). The window size was set to 200 nt and the step size to 20 nt.

## Results

### Distribution of sample sources

**Figure 1:**
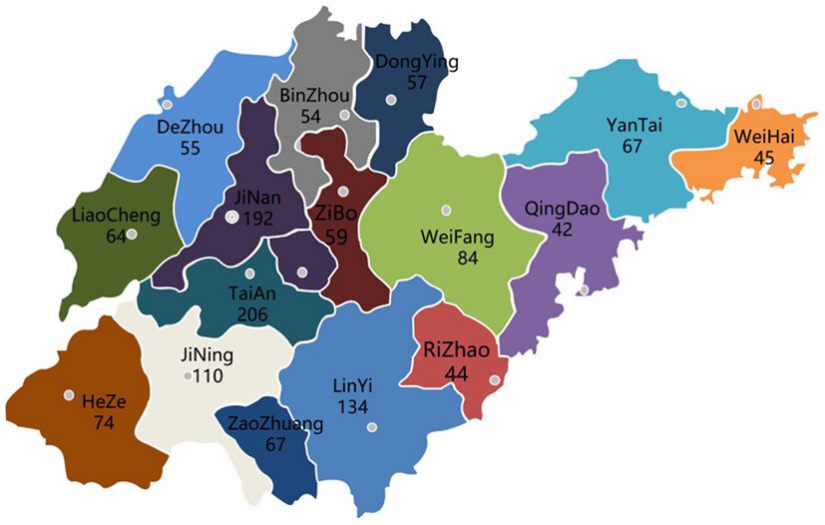
Map of sample collection and distribution in Shandong Province from 2020 to 2021 (the number represents the number of samples collected).

A total of 1,344 suspected PRRS-positive samples were collected from all cities in Shandong Province in 2020–2021. The number of samples in different regions is shown in Figure 1.

### RT-PCR results

**Figure 2:**
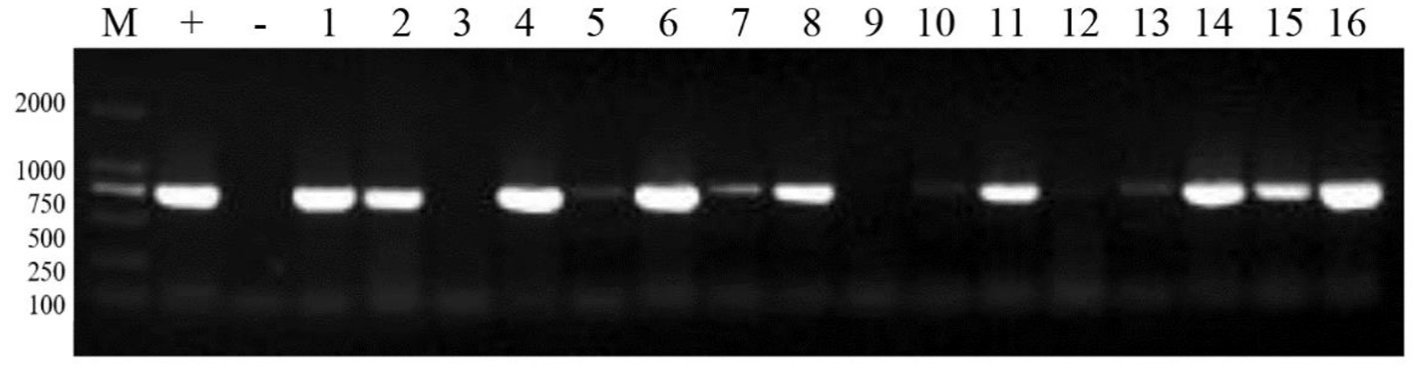
PCR results of some PRRSV positive samples. M: 2,000 marker, +: positive control, –: negative control, 1–16: sample numbers.

Total RNA was extracted from cultured virus fluid and reverse-transcribed into cDNA. PCR was performed using cDNA as the template. The results indicated that the number of PRRSV positive samples was 342, and the detection rate was 25.44%. PCR results from selected PRRSV-positive samples are presented in Figure 2.

### Genetic evolutionary analysis of ORF5 gene

**Figure 3:**
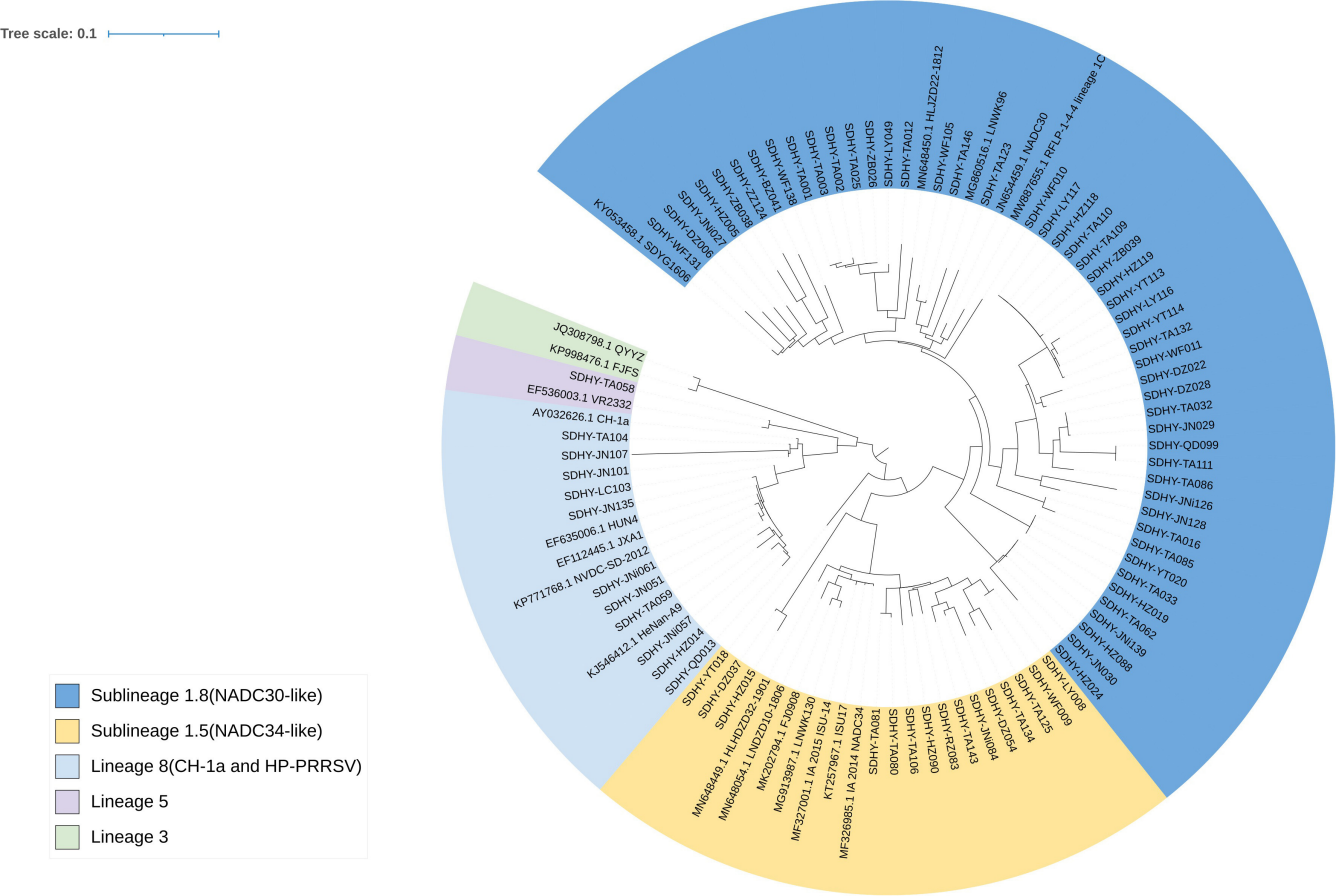
Phylogenetic tree based on the PRRSV ORF5 sequence. Evolutionary analysis of maximum likelihood performed in MEGA X. Multiple sequence alignments generated using Clustal W. ITOL was used to modify the genetic evolutionary tree, using different colors to distinguish different lineages and reference strains with GenBank sequence numbers.

In all, 76 strains were sequenced for the PRRSV ORF5 gene (Figure 3). Among them, 49 strains belonged to Sublineage 1.8 in the evolutionary genetic map of the ORF5 gene, representing 64.47% of isolates. Fifteen strains (19.74%) were classified as Sublineage 1.5. One strain (1.32%) was classified as Sublineage 5. Eleven strains (14.47%) were classified as Lineage 8.

### Mutation analysis of the deduced amino acid site of ORF5 gene

**Figure 4:**
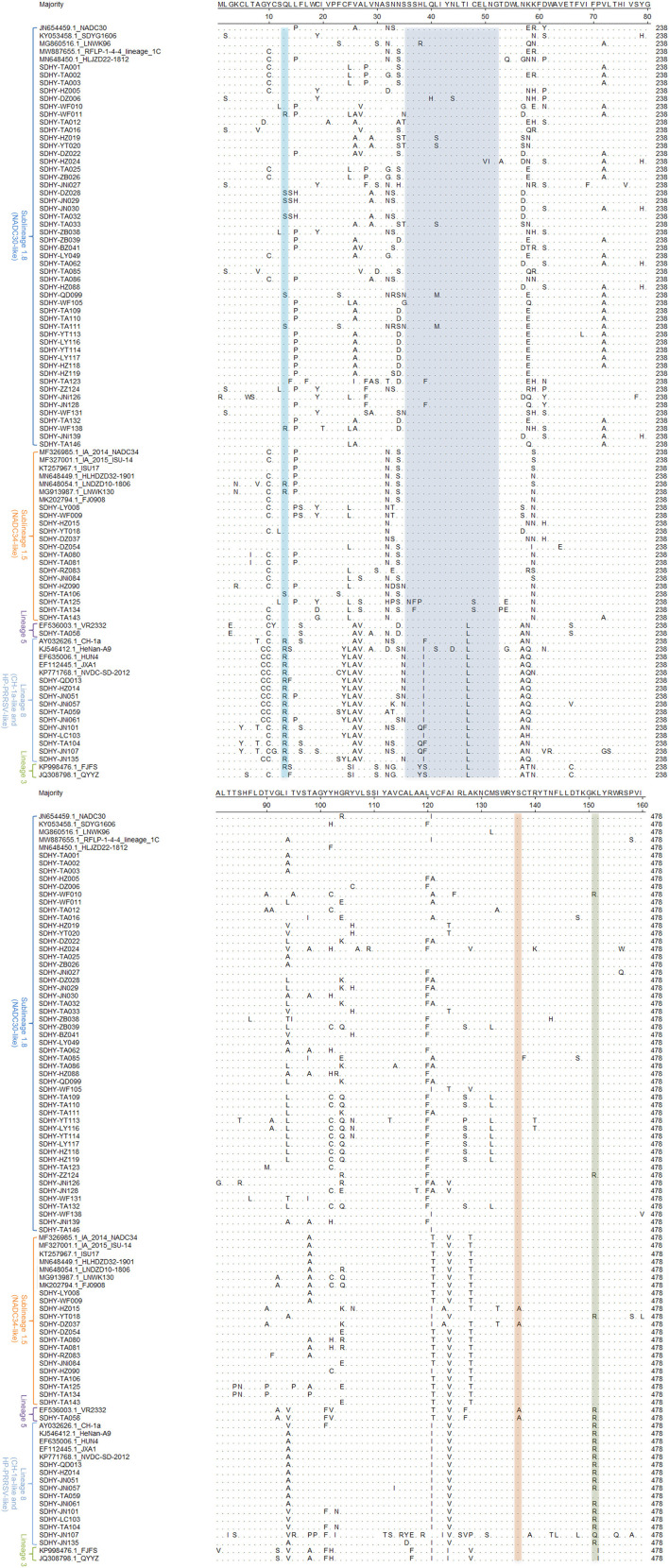
Analysis based on the major amino acid mutation sites of the PRRSV ORF5 gene.

The MegAlign module of DNASTAR Lasergene software was used to analyze the deduced amino acid mutation sites of 76 ORF5 genes in this study in comparison to some of the reference strains (Figure 4). A few typical mutations have been found. Amino acids 13 and 151 of ORF5-encoding GP5 protein were associated with virulence-related sites of the virus. Virulent strains of R^13^ and R^151^ are those that are often highly virulent (37–39). In this experiment, 13 strains of virus exhibited a Q^13^ → R^13^ mutation. Two strains were NADC30-like and the remaining 11 strains were CH-1a and HP-PRRSV-like.

Thirteen strains showed the K^151^ → R^151^ mutation, including two strains that were NADC30-like. One strain was NADC34-like and one strain was VR2332-like. The other nine strains were CH-1a and HP-PRRSV-like.

Mutations in the neutralizing epitope region at positions 36–52 of the amino acids encoded by ORF5 may cause the virus to escape the neutralizing effect induced by vaccine immunity. Reduce the protection effectiveness of vaccine immunity. In this study, multiple strains were isolated with mutations in the neutral epitope region. Twenty-three strains showed mutations in the neutral epitope region, of which 9 strains were NADC30-like, 2 strains were NADC34-like, 1 strain was VR2332-like, and the other 11 strains were CH-1a and HP-PRRSV-like.

Amino acid 137 (A^137^) of GP5 is unique to the VR2332, MLV, and RespPRRS/Repro vaccine strains and is considered to be a discriminating site between wild strains and vaccine strains (38, 40, 41). In this study, three isolates showed mutations from S^137^ to A^137^. Two of these were NADC34-like and one was VR2332-like.

### Recombination analysis

**Figure 5:**
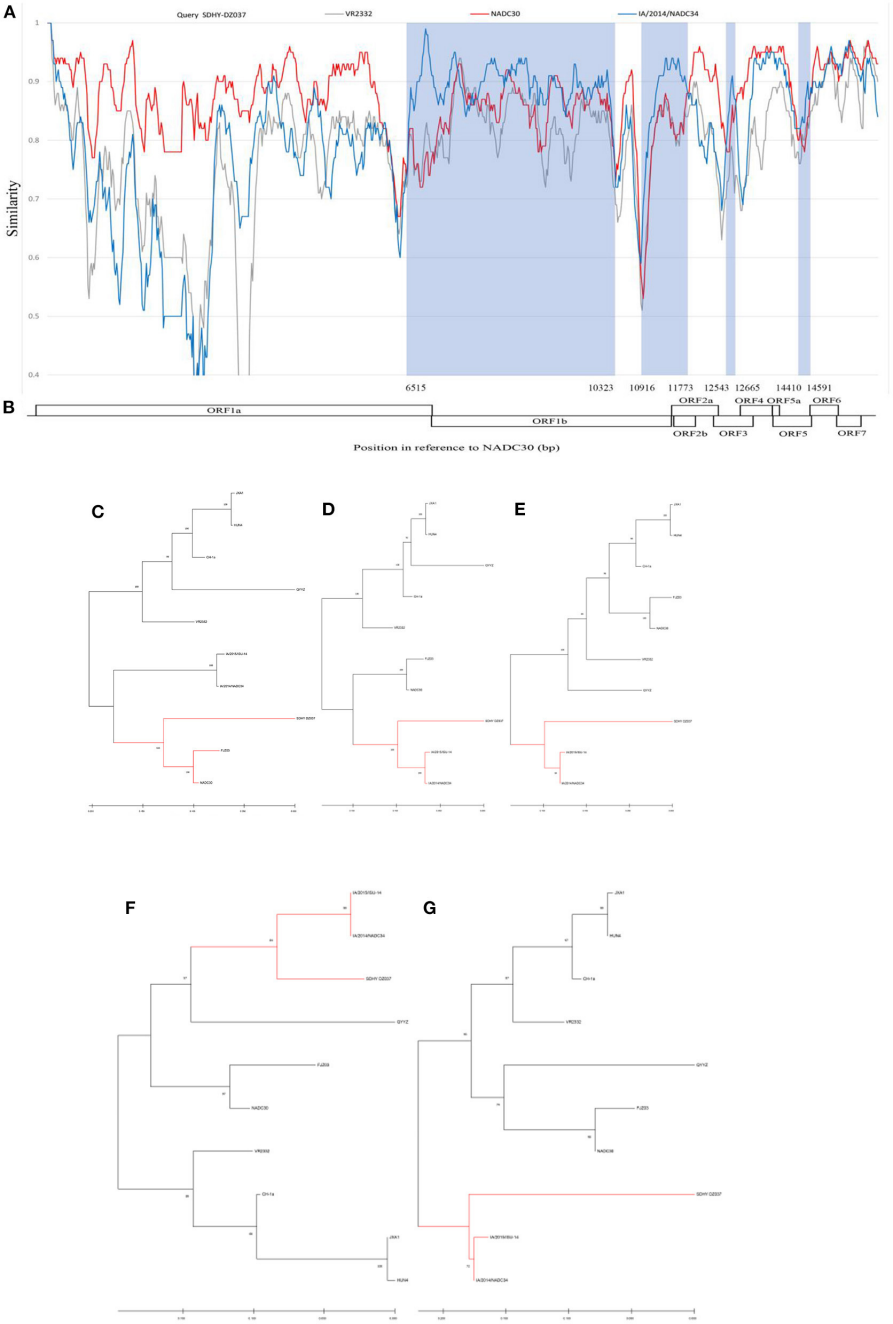
In this study, the PRRSV representative strains and SDHY-DZ037 were selected for genome-wide recombination analysis. Similarity maps were generated by the SimPlot v3.5 software **(A)**. The gene region corresponding to the SDHY-DZ037 recombination was shown with NADC30 (GenBank: JN654459.1) **(B)**. Phylogenetic tree based on full-length genome sequence **(C)**. Phylogenetic tree of the nucleotide recombination region at positions 6,515–10,323 **(D)**. Phylogenetic tree of the nucleotide recombination region at positions 10,916–11,773 **(E)**. Phylogenetic tree of the nucleotide recombination region at positions 12,543–12,665 **(F)**. Phylogenetic tree of the nucleotide recombination region at positions 14,410–14,591 **(G)**. Evolutionary analysis of maximum likelihood performed in MEGA X. Multiple sequence alignments generated using Clustal W.

In this study, we found that both SDHY-HZ015 and SDHY-DZ037 showed A^137^. A^137^ is generally considered a unique locus of the VR2332, MLV, and RespPRRS/Repro vaccine strains. We therefore conducted a genome-wide recombination analysis, using the HiScript III 1st Strand cDNA Synthesis Kit (Vazyme). The resultant total RNA was reverse transcribed into the cDNA, according to the instructions. PCR amplification was performed using the primers of Supplementary Table 1 (42). The results were observed through agarose gel electrophoresis under a gel system imager. We sent RT-PCR positive products for sequencing (BGI Genomics). Finally, an entire genome of SDHY-DZ037 was successfully isolated. Representative strains of each PRRSV strain were selected as reference. Recombination events and recombination breakpoints were confirmed through RDP4 software. The results are in Supplementary Table 2. Validation and presentation of results was done using SimPlot (Figure 5A). SDHY-DZ037 was a recombinant NADC30-like PRRSV and NADC34-like PRRSV. The primary parent strain was NADC30-like, and the secondary parent strain was NADC34-like. Four recombination events were identified by the RDP4 and SimPlot software. The results showed that: recombination event 1 occurred at nucleic acid 6515-10323 nt (Figure 5D); event 2 occurred at nucleic acid 10916-11773 nt (Figure 5E); event 3 occurred at nucleic acid 12543-12665 nt (Figure 5F); event 4 occurred at nucleic acid 14410-14591 nt (Figure 5G). The area of recombinant gene was shown in the NADC30 (GenBank: JN654459.1) genome (Figure 5B). A phylogenetic analysis was performed on the entire genome and each recombinant region. The genome-wide phylogenetic tree showed that SDHY-DZ037 belonged to Sublineage 1.8 (NADC30-like) (Figure 5C), and all the recombinant regions belonged to Sublineage 1.5 (NADC34-like).

## Discussion

Since the emergence of PRRSV in China, PRRS has caused serious harm to the country's swine industry. The positive rate of PRRSV in Shandong Province was 9.58% in 2018–2019, and primarily Lineage 1 was predominant (43). To understand the prevalence of PRRSV in Shandong Province in 2020–2021, a total of 1,344 samples (mainly blood and nasal swabs from suspected sick pigs) were collected from pig farms of various sizes. The samples came from a wide array of sources, mainly Tai'an and the surrounding cities, and has covered pig farms of different scales in all cities of Shandong Province. A total of 342 samples were positive for PRRSV, giving a positive rate of 25.44%, showing that PRRSV is still one of the main pathogens threatening the swine industry in Shandong. Overall, 76 strains were isolated, of which 49 strains were NADC30-like, accounting for 64.47% of the isolated viruses. Fifteen strains were NADC34-like, representing 19.74% of the isolated viruses; 11 strains were CH-1a and HP-PRRSV-like, accounting for 14.47% of the isolated viruses; and 1 strain was VR2332-like and accounted for 1.32% of the isolated viruses. The NADC30-like strains remain the dominant strains in Shandong. This corresponds to the previous study (43).

The NADC34-like strain was first discovered in Liaoning in 2017 (18). To date, the prevalence of the NADC34-like strain has been reported in at least 10 provinces, including Heilongjiang, Liaoning, Henan, Hebei, Fujian, Jiangsu, Sichuan, Tianjin, and Shandong (24, 44). However, the prevalence of NADC34-like in Shandong Province is still unknown. We conducted an epidemiological survey of PRRSV in Shandong in 2020–2021. The study found that in 2020, NADC30-like strains accounted for 75.00% of PRRSV-positive samples and NADC34-like strains accounted for 15%. In 2021, NADC30-like strains accounted for 52.78% of PRRSV-positive samples and NADC34-like strains accounted for 25%. This indicates that the NADC34 strain is starting to show an epidemic trend in Shandong. One assumes that it could become the dominant strain in the years to come.

Analysis comparing the deduced amino acid loci of the ORF5 gene in the isolated strains and in some reference strains showed that the mutation Q^13^ → R^13^ was present in all viruses of Lineage 8, while the mutation K^151^ → R^151^ was absent in two viruses at amino acid position 151. SDHY-TA059 still had K at amino acid position 151, and SDHY-JN107 had the mutation K^151^ → Q^151^. It appears that some of the strong strains may have lost some of their strong virulence characteristics during the evolutionary process, thereby weakening their virulence (37). Two strains of Sublineage 1.8 showed mutations of Q^13^ → R^13^, as opposed to other strains. In addition, two strains of Sublineage 1.8, one strain of Lineage 5, and one strain of Sublineage 1.5 had mutations of K^151^ → R^151^. This indicated that certain mutations occur on specific sites during the continuous mutation of viruses. This may make it more virulent than other viruses of the same type. Mutations in the neutralizing epitope region may cause the virus to be insensitive to the neutralizing effect of vaccination and thus avoid it. In this study, 23 isolated strains showed mutations in the neutralizing region of the epitope, accounting for 30.26% of the isolates. These strains may be insensitive to vaccine immunization, which may be an important reason for the poor efficacy of the current PRRSV vaccine.

The S^137^ → A^137^ mutation occurred in three of the isolated strains, two of which were NADC34-like and one VR2332-like. It has been reported that amino acid 137 of GP5 was serine for wild strains and alanine for VR2332, MLV, and RespPRRS/Repro vaccine strains (38, 40, 41). Some scholars believe that A^137^ can be used to distinguish Lineage 5 and Lineage 1 (45). Among all the strains isolated in this study, the amino acid 137 of GP5 was mutated to alanine in three strains. The other strains were serine. The three mutated viruses are SDHY-HZ015, SDHY-DZ037, and SDHY-TA058. In this study, a genome-wide recombination of SDHY-DZ037 was analyzed by RDP4 and SimPlot software. The results showed that SDHY-DZ037 was found to be a recombinant strain of NADC30-like and NADC34-like strains but without recombinant VR2332. We speculated that perhaps amino acid position 137 of GP5 had mutated during the PRRSV epidemic. Therefore, perhaps A^137^ was no longer a specific amino acid site within VR2332, MLV, and RespPRRS/Repro vaccine strains, and A^137^ may no longer be suitable for distinguishing Lineage 1 and Lineage 5.

## Conclusions

In summary, the NADC30-like strain remained the dominant epidemic strain of PRRSV in Shandong, China, in 2020–2021. It should be noted that NADC34-like strains have also been quite prevalent in this area. Some isolates had mutations in amino acids 13 and 151 of ORF5 and in the region of the neutralizing epitope (aa36–aa52), which may alter the virulence and increase the virus's ability to escape immunity.

## Data availability statement

The datasets presented in this study can be found in online repositories. The names of the repository/repositories and accession number(s)can be found below: https://www.ncbi.nlm.nih.gov/genbank/, ON890168-ON890242 and OP168793.

## Ethics statement

The animal study was reviewed and approved by the Animal Ethics Committee of Shandong Agricultural University.

## Author contributions

Writing–original draft preparation was done by PL and YS. Writing–review and editing was done by BL, ML, and FM. Software was done by PL and YL. Investigation was done by JL, YZ, and TW. Funding acquisition was done by SL. All authors have read and agreed to the published version of the manuscript.

## Funding

This study was funded by the Shandong Provincial Agricultural Major Application Technology Innovation Project (Establishment and Demonstration of Healthy Pig Breeding Technology Integration and Product Supply Chain Traceability System).

## Conflict of interest

Author FM was employed by Huayun (Shandong) Inspection and Quarantine Service Co., Ltd. The remaining authors declare that the research was conducted in the absence of any commercial or financial relationships that could be construed as a potential conflict of interest.

## Publisher's note

All claims expressed in this article are solely those of the authors and do not necessarily represent those of their affiliated organizations, or those of the publisher, the editors and the reviewers. Any product that may be evaluated in this article, or claim that may be made by its manufacturer, is not guaranteed or endorsed by the publisher.
