# Supplementary material for: Epidemiological survey of PRRS and genetic variation analysis of the ORF5 gene in Shandong Province, 2020–2021
Source: Front Vet Sci. 2022 Sep 15;9:987667. doi: 10.3389/fvets.2022.987667 (PMC9521713; doi:10.3389/fvets.2022.987667)
Supplement: Supplementary file 1 [file Data_Sheet_1.PDF]

Supplementary Table 1: Primers used in this experiment to isolate the full length

Names*	Primer Sequence (5'-3')	Length (bp)
NADC30-I-F	ATGACGTATAGGTGTTGGCTCTATGC	2168
NADC30-I-R	AGCTTTCTCAAGCCTAGCCAAGC	
NADC30-II-1-F	GGGTTTGACCCTGCCTGCCTTGA	1941
NADC30-II-1-R	AAACTCACAAGCAGTGCCGACTG	
NADC30-II-2-F	CTGCTGGCTGGCTTTTGCTGTTG	2148
NADC30-II-2-R	CCTCCTTCCAGTTCGGGTTTGGC	
NADC30-III-F	CGCCCTTCAGGCCAGTTTTGTAA	2157
NADC30-III-R	CGCTAGGGGTCTTGTAAGGTATGTC	
NADC30-IV-F	AGGGGCAACCCTGAACGGGTAAAAG	2199
NADC30-IV-R	AAGCCTCAAGACATCAAGATGATTG	
NADC30-V-F	AAAGCTTTGGGAACGTGTCGGTTTA	2109
NADC30-V-R	AACGGCAGAGCGCGCACGGAGTATC	
NADC30-VI-F	CATCGCCGGATGGTTGGTGGTACTT	1747
NADC30-VI-R	GCCATTCAGCTCACATATCGTCAGG	
NADC30-VII-F	GATATGTTGGGGAAATGCTTGACCG	1627
NADC30-VII-R	TTAATTACGGCCGCATGGTTCTC	
NADC34-I-F	ATGACGTATAGGTGTTGGCTCTATGC	2168
NADC34-I-R	AGCTTTCTCAAGCCTAGCCAAGCATT	
NADC34-II-1-F	GCAAAGATTGACTCGTACCTTCG	1941
NADC34-II-1-R	AGCTCAAAAGAGTGAAGGATGTC	
NADC34-II-2-F	TTGTTGGTTGGCTTTCGCTGTTG	2148
NADC34-II-2-R	CAAGCAAGGCACAAAGATCAGAA	
NADC34-III-F	CGCCCTTCAGGCCAGTTTTGTAA	2157
NADC34-III-R	CACTAGGGGTCTTGTAAGGTATGTC	
NADC34-IV-F	AATACAGGCTTGTGACATTAGGC	2199
NADC34-IV-R	CATCCTGAACCTGTTGGAGGAGC	
NADC34-V-F	CAGCACCGCGTTACTTCCAACTT	2109
NADC34-V-R	CACCCATTTCATTTCAGTCCAGG	
NADC34-VI-F	GTTCTCGCTAGACGACCCAGTCA	1747
NADC34-VI-R	TCTGTGCCATTCAGCTCACATAT	
NADC34-VII-F	GATATGTTGGGGAAATGCTTGACCG	1627
NADC34-VII-R	TTAATTACGGCCGCATGGTTCTC	

F represents forward PCR primer; R represents reverse PCR primer.
