# Supplementary material for: Epidemiological survey of PRRS and genetic variation analysis of the ORF5 gene in Shandong Province, 2020–2021
Source: Front Vet Sci. 2022 Sep 15;9:987667. doi: 10.3389/fvets.2022.987667 (PMC9521713; doi:10.3389/fvets.2022.987667)
Supplement: Supplementary file 2 [file Data_Sheet_2.PDF]

Information on recombination events of QYYZ-like PRRSV isolates detected by RDP4 software

Isolates	Major parental strain	Minor parental strain	Breakpoints		Average p-value of the detection methods						
			Beginning	Ending	RDP	GENECONV	BootScan	MaxChi	Chimera	SiScan	3Seq
SDHY-DZ037	NADC30	IA/2014/NADC34	6515	10323			$4.121*10^{-20}$	$6.952*10^{-21}$	$7.555*10^{-22}$		$2.220*10^{-16}$
	NADC30	IA/2014/NADC34	10916	11773	$4.519*10^{-17}$		$7.583*10^{-15}$	$1.311*10^{-12}$	$7.205*10^{-15}$		$2.220*10^{-16}$
	NADC30	IA/2014/NADC34	12543	12665	$6.772*10^{-10}$	$2.033*10^{-08}$	$9.080*10^{-10}$	$1.269*10^{-05}$	$6.081*10^{-04}$		$4.211*10^{-05}$
	NADC30	IA/2014/NADC34	14410	14591	$9.600*10^{-04}$	$3.780*10^{-03}$	$2.666*10^{-03}$	$7.100*10^{-03}$	$7.930*10^{-03}$		
